# Management of Intrathoracic Benign Schwannomas of the Brachial Plexus

**DOI:** 10.1155/2014/130492

**Published:** 2014-07-22

**Authors:** Alessandro Bandiera, Giampiero Negri, Giulio Melloni, Carlo Mandelli, Simonetta Gerevini, Angelo Carretta, Paola Ciriaco, Armando Puglisi, Piero Zannini

**Affiliations:** ^1^Department of Thoracic Surgery, San Raffaele Scientific Institute, Via Olgettina 60, 20132 Milan, Italy; ^2^Department of Neurosurgery, San Raffaele Scientific Institute, Via Olgettina 60, 20132 Milan, Italy; ^3^Department of Neuroradiology, San Raffaele Scientific Institute, Via Olgettina 60, 20132 Milan, Italy

## Abstract

Primary tumours of the brachial plexus are rare entities. They usually present as extrathoracic masses located in the supraclavicular region. This report describes two cases of benign schwannomas arising from the brachial plexus with an intrathoracic growth. In the first case the tumour was completely intrathoracic and it was hardly removed through a standard posterolateral thoracotomy. In the second case the tumour presented as a cervicomediastinal lesion and it was resected through a one-stage combined supraclavicular incision followed by left video-assisted thoracoscopic surgery. A brachial plexus tumour should be suspected not only in patients with a supraclavicular or cervicomediastinal mass but also in those with intrathoracic apical lesions. A preoperative magnetic resonance imaging study of brachial plexus should be performed in such cases in order to plan the correct surgical approach.

## 1. Introduction

Primary tumours of the brachial plexus are rare entities and they are most commonly represented by schwannomas and neurofibromas. Schwannomas arising from the brachial plexus are usually extrathoracic tumours located in the supraclavicular region [1, 2]. An intrathoracic growth of these tumours is extremely rare and only few cases have been reported in the literature. [3, 4].

This paper describes two different presentations of benign intrathoracic brachial plexus schwannoma and their surgical treatment.

## 2. Case Presentation

### 2.1. Case 1

**Figure 1:**
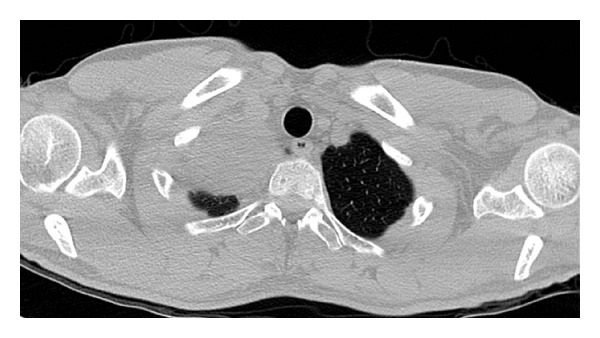
CT scan (CT) (axial view) showing a well-circumscribed, encapsulated ovoid mass, measuring approximately 5 cm, located in proximity to the right thoracic outlet.

A 45-year-old man was admitted to our department of thoracic surgery for a suspected intrathoracic tumour which was found by chance after a chest radiograph for a persistent cough. Subsequent computed tomographic (CT) scan showed a well-circumscribed, ovoid mass, measuring approximately 5 cm, located close to the right thoracic outlet (Figure 1, axial view). Differential diagnosis included a neurogenic thoracic lesion and a primitive lung cancer. The patient was submitted to surgery for diagnostic and therapeutic purposes.

**Figure 2:**
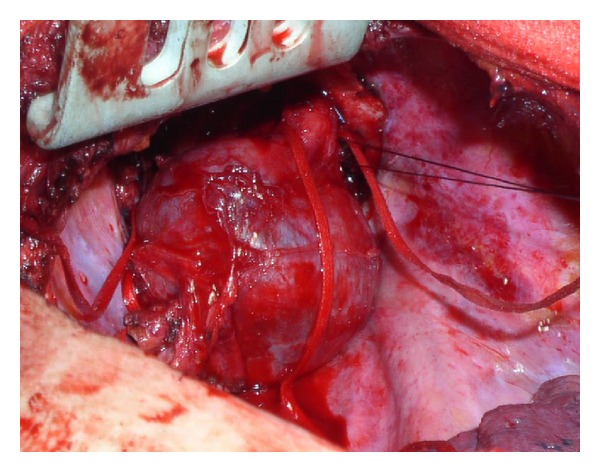
Intraoperatively, the tumour was found to originate from the posterior cord of the brachial plexus.

At preliminary thoracoscopy the tumour was found to originate from the apical chest wall. The procedure was converted into an open standard posterolateral thoracotomy due to the apical site of the tumour and its volume. During the dissection manoeuvres, the tumour was found to originate from the posterior cord of the brachial plexus (Figure 2). Tumour resection was performed, in collaboration with neurosurgeons, with the aid of operative microscope and electrophysiological monitoring to spare functioning motor fibres. Intraoperative stimulation of the functioning motor fibres gave rise to a recordable evoked response while the stimulation of the tumour evoked no responses. Functioning motor fibres could be recognised and the tumour could be separated from the nerve fibres. Therefore, the resection of the tumour could be performed with sparing of the entire nerve.

Pathological examination revealed a benign schwannoma. The postoperative course was uneventful and the postoperative neurological status showed no deficit.

### 2.2. Case 2

**Figure 3:**
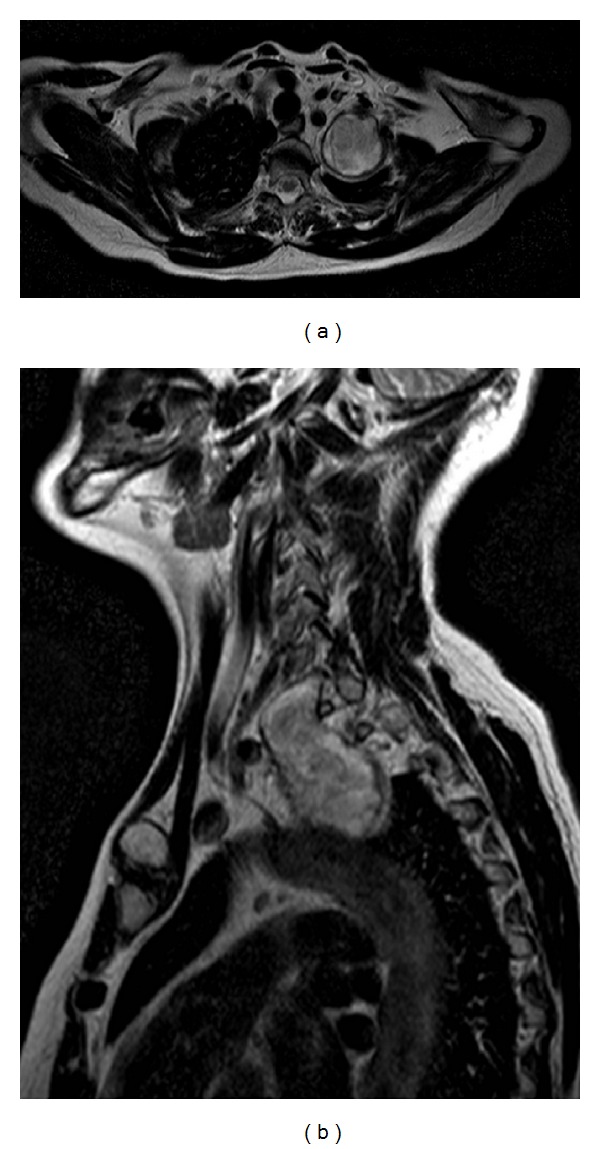
((a) and (b)) Axial and sagittal view. MRI of brachial plexus showing a well-circumscribed encapsulated tumor arising from the left brachial plexus.

A 48-year-old female was admitted to our department of thoracic surgery for a left apical lesion which was found by chance after a routine chest X-ray. The CT scan and magnetic resonance imaging (MRI) of the brachial plexus showed a well-circumscribed tumour located in the superior posterior mediastinum with extension into lower cervical region (Figures 3(a) and 3(b), axial and sagittal view). The mass measured 6.5 cm × 4.5 cm. A dedicated brachial plexus MRI protocol clearly showed the neural origin of the tumour, without sign of infiltration and with an extraclavicular growth into thoracic region.

**Figure 4:**
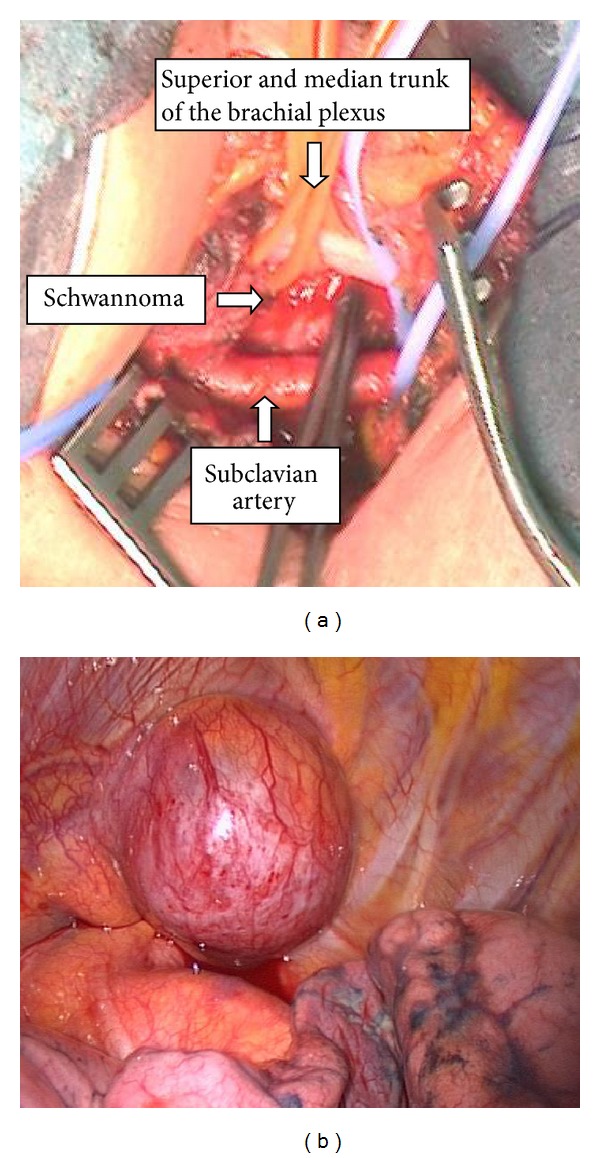
((a) and (b)) Cervical and videothoracoscopic access. The surgical approach was defined as a one-stage combined supraclavicular incision (a) followed by left video-assisted thoracoscopic surgery (b).

The surgical approach was defined as a one-stage combined supraclavicular incision followed by left video-assisted thoracoscopic surgery (VATS) (Figure 4). The supraclavicular incision permitted an accurate and safe exposure of the cervical portion of the tumour and its mobilization from the superior and median trunk of the brachial plexus and from the subclavian artery. The patient was then placed on the right lateral decubitus and dissection of the tumour from brachial plexus inferior trunk and its en bloc removal from the chest were performed through a left VATS with a small (6 cm) auxiliary thoracotomy. The postoperative course was uneventful with only a transient mild strength deficit in the left hand. Pathological examination revealed a benign schwannoma.

## 3. Discussion

Schwannomas are the most common intrathoracic neurogenic tumours. They generally involve the posterior mediastinum arising from an intercostal nerve or a sympathetic chain. These tumours arise from the cells of the nerve sheath usually as single benign lesions in the costovertebral sulcus.

Schwannomas of the brachial plexus are rare entities and they generally present as extrathoracic tumours located in the supraclavicular region [1, 2]. An intrathoracic growth of this type of tumours is extremely rare and only few cases have been reported in the literature [3, 4].

This report describes two cases of intrathoracic growth of benign schwannoma arising from the brachial plexus. In our opinion, the explanation for this pattern of intrathoracic growth could be that these tumours, originating from the lowest cord of the brachial plexus, were attracted into the chest cavity by the negative intrathoracic pressure.

In the first case, the neural origin of the tumour from the brachial plexus was not suspected and the preoperative evaluation was incomplete as a MRI with a dedicated brachial plexus protocol was not performed. During the operation, however, the tumour was found to originate from the posterior cord of the brachial plexus. Resection of the mass was hampered by the thoracic surgical approach and could be performed only through a standard posterolateral thoracotomy and with the aid of an operative microscope and electrophysiological monitoring.

In the second case, the localization of the tumour and its neural relationship were precisely identified by a correct MRI protocol for the study of brachial plexus and thoracic outlet [5]. This finding allowed us to plan a safe one-stage combined supraclavicular approach followed by left VATS. Through the cervical incision, the tumour could be safely manipulated away from the nervous and vascular structures of the thoracic outlet. Once the tumour was mobilized from the neck, only a mini-invasive thoracic procedure, in contrast to the open access of the first case, was performed to dissect it from the upper mediastinum and to remove it.

In conclusion, brachial plexus tumours are rare entities usually presenting as supraclavicular lesions. On the basis of our experience, however, a brachial plexus tumour should be suspected not only in patients with a supraclavicular mass and with cervicomediastinal lesions but also in those with intrathoracic apical tumours. Therefore, a correct MRI study of the thoracic outlet should be performed for a more precise evaluation of this region and its anatomical structures. A correct preoperative identification of the origin from the brachial plexus of these tumours allows planning their surgical resection. In our opinion, a combined supraclavicular approach followed by a limited thoracic access is the procedure of choice. This allows avoiding standard open thoracic approaches and the surgeons are able to manage for combined surgical teams since the preoperative period.
